# Covalent narlaprevir- and boceprevir-derived hybrid inhibitors of SARS-CoV-2 main protease: room-temperature X-ray and neutron crystallography, binding thermodynamics, and antiviral activity

**DOI:** 10.21203/rs.3.rs-1318037/v1

**Published:** 2022-02-11

**Authors:** Daniel Kneller, Hui Li, Gwyndalyn Phillips, Kevin Weiss, Qiu Zhang, Mark Arnould, Colleen Jonsson, Surekha Surendranathan, Jyothi Parvathareddy, Matthew Blakeley, Leighton Coates, John Louis, Peter Bonnesen, Andrey Kovalevsky

**Affiliations:** Oak Ridge National Laboratory; Oak Ridge National Lab; Oak Ridge National Laboratory; Oak Ridge National Laboratory; Oak Ridge National Laboratory; Oak Ridge National Lab; University of Tennessee Health Science Center; University of Tennessee Health Science Center; University of Tennessee Health Science Center; Institut Laue-Langevin; Oak Ridge National Laboratory; The National Institutes of Health; Oak Ridge National Laboratory; Oak Ridge National Laboratory

## Abstract

The COVID-19 pandemic continues to disrupt everyday life, with constantly emerging SARS-CoV-2 variants threatening to render current vaccines ineffective. Small-molecule antivirals can provide an important therapeutic treatment option that is subject to challenges caused by the virus variants. The viral main protease (M^pro^) is critical for the virus replication and thus is considered an attractive drug target for specific protease inhibitors. We performed the design and characterization of three reversible covalent hybrid inhibitors BBH-1, BBH-2 and NBH-2, whose structures were derived from those of hepatitis C protease inhibitors boceprevir and narlaprevir. A joint X-ray/neutron structure of the M^pro^/BBH-1 complex demonstrated that a Cys145 thiolate reaction with the inhibitor’s keto-warhead creates a negatively charged oxyanion, similar to that proposed for the M^pro^-catalyzed peptide bond hydrolysis. Protonation states of the ionizable residues in the M^pro^ active site adapt to the inhibitor, which appears to be an intrinsic property of M^pro^. Structural comparisons of the hybrid inhibitors with PF-07321332 revealed unconventional interactions of PF-07321332 with M^pro^ which may explain its more favorable enthalpy of binding and consequently higher potency. BBH-1, BBH-2 and NBH-2 demonstrated comparable antiviral properties *in vitro* relative to PF-07321332, making them good candidates for further design of improved antivirals.

## Introduction

COronaVIrus Disease 2019 (COVID-19), caused by the novel severe acute respiratory syndrome coronavirus 2 (SARS-CoV-2), unknown to the world just over two years ago, continues to ravage the human population with wave after wave. The COVID-19 pandemic has had a catastrophic impact on human life and well-being, with over 350 million confirmed cases and over 5.6 million deaths to date globally and with millions of people suffering from long COVID complications (Sato et al., 2021; Nagu et al., 2021; Nalbandian et al., 2021). While vaccines are now readily available in some countries to protect from the disease (Meo et al., 2021), a substantial proportion of the population remains unvaccinated due to various factors, including an inability to vaccinate due to preexisting conditions or unwillingness due to personal views. This situation promotes, at least in the near future, the selection of new SARS-CoV-2 variants with multiple mutations and thus threatens the effectiveness of existing vaccines (Bian et al., 2021; Levine-Tiefenbrun et al., 2021; Wang et al., 2021). Small-molecule antiviral drugs targeting viral proteins essential for the virus replication can help alleviate this problem as the mechanism of action of such drugs would not be affected by mutations in the spike protein that would normally arise in the vaccine escape SARS-CoV-2 variants. However, repurposing of known clinical drugs for the treatment of COVID-19 has not met with much success (WHO solidarity trial consortium, 2021; Riva et al., 2020; Beigel et al., 2020). Therefore, design and development of specific antivirals against SARS-CoV-2 remains an urgent and pressing matter.

Among the initial steps in the virus replication cycle, the proteolytic function of the 3-chymotrypsin-like protease (3CL^pro^, or main protease, M^pro^) is vital for SARS-CoV-2 proliferation. After translation of the viral genomic mRNA by the host ribosomes, M^pro^ cuts the resulting large polyproteins pp1a and pp1ab into nonstructural proteins that assemble into the replication-transcription complex (Wu et al., 2020; Xu et al., 2020). Thus, inhibition of the action of M^pro^ impairs viral replication. Accordingly, there has been a significant increase in research activity over the past two years to create specific M^pro^ inhibitors with potential therapeutic applications (Zhang et al., 2020; Dai et al., 2020; Ullrich & Nitsche, 2020; Rathnayake et al., 2020; Ghosh et al., 2020; Hoffman et al., 2020; Hattori et al., 2021). Historically, design, development and clinical usage of HIV-1 and hepatitis C virus protease inhibitors (Ghosh et al., 2016; Liverton, 2019) informs us that multiple continuously improving clinical drugs will be required to overcome various challenges when treating viral infections.

M^pro^ is a cysteine protease (Gorbalenya & Snijder, 1996; Tong, 2002) that hydrolyzes peptide bonds by utilizing a noncanonical catalytic dyad comprised of Cys145 and His41 and an oxyanion hole formed by the main-chain amide NH groups of Gly143, Ser144 and Cys145. The oxyanion hole evolved to stabilize the negatively charged tetrahedral intermediates along the M^pro^-catalyzed reaction pathway. Furthermore, catalysis is believed to be assisted by a conserved water molecule that is hydrogen bonded to His41, His164 and Asp187, which together with Arg40 and Tyr54 comprise a partially negatively charged cluster thought to act as the third catalytic residue (Wang et al., 2020). The M^pro^ active site can fit substrates or inhibitors containing chemical groups at positions P5 through P3 to occupy substrate binding subsites S5-S3 (Kneller et al., 2021a; MacDonald et al., 2021). Subsites S1 and S2 are the most selective. S1 exclusively binds Gln, whereas S2 prefers medium-sized hydrophobic residues like Leu or Phe (Zhu et al., 2011; Zhang et al., 2020). Two major strategies have been employed to inhibit M^pro^ efficiently. On the one hand, M^pro^ inhibitors can be noncovalent, binding to the enzyme active site only through hydrogen bonding and hydrophobic interactions. Conversely, the nucleophilic nature of Cys145 thiolate can be exploited utilizing reactive electrophilic groups known as warheads that form an additional covalent bond with the enzyme improving inhibitor binding affinity relative to a noncovalent molecule. For example, several research groups have focused their attention on structure-based drug design of noncovalent M^pro^ inhibitors starting from the anti-epileptic drug perampanel (Deshmukh et al., 2021; Zhang et al., 2021), or from ML188 and ML300 previously designed to inhibit SARS-CoV-1 M^pro^ (Jacobs et al. 2013; Turlington et al., 2013; Kitamura et al. 2021; Han et al., 2021). We have recently performed a structure-activity relationship study (Kneller et al., 2021b) on a noncovalent compound newly discovered by high-throughput virtual screening (Clyde et al., 2021). Others have designed and evaluated a series of peptidomimetic reversible or irreversible covalent inhibitors (Konno et al., 2021; Bai et al., 2021; Yang et al., 2021), or nonpeptidic small molecules with a novel chemical scaffold (Ghosh et al., 2021).

**Figure 1:**
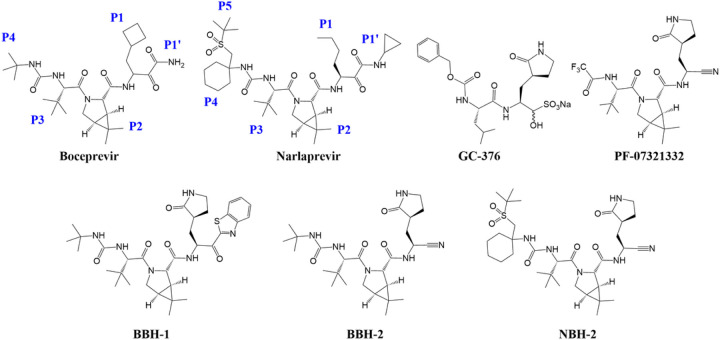
Chemical structures of protease inhibitors.

Hepatitis C virus clinical protease inhibitor boceprevir (Victrelis™, Merck, Venkatraman, 2019) and the feline peritonitis virus protease inhibitor GC-376 (Figure 1), both peptidomimetic reversible covalent inhibitors, demonstrated effective binding and inhibition of SARS-CoV-2 M^pro^, and robust *in vitro* antiviral activity (Ma et al., 2020; Fu et al., 2020; Kneller et al. 2020a; Oerlemans et al., 2021). GC-376 is a prodrug that turns into the reactive aldehyde in aqueous solution. Not surprisingly, the chemical structures of boceprevir and GC-376 were combined to create hybrid inhibitors with improved *in vitro* and *in vivo* antiviral properties (Xia et al., 2021; Qiao et al., 2021). Researchers from Pfizer followed a similar strategy by additionally incorporating functional groups from compounds previously designed to inhibit SARS-CoV-1 M^pro^ (Thanigaimalai et al., 2013; Konno et al., 2013). Pfizer’s strategy ultimately resulted in the design and development of an oral drug PF-07321332 (nirmatrelvir, Figure 1, Owen et al., 2021) that has shown excellent efficacy in preventing hospitalization and death (https://clinicaltrials.gov/ct2/show/NCT04960202). Paxlovid™ (PF-07321332/ritonavir) was recently approved by the U.S. Food and Drug Administration for emergency use to treat COVID-19 patients. Building from our recent hepatitis C virus protease inhibitor repurposing work (Kneller et al., 2020a), we have followed an analogous strategy, working independently from and in parallel with the other research groups, to design hybrid reversible covalent inhibitors of M^pro^ that combine the chemical features of boceprevir and narlaprevir (Arlansa™, R-Pharm, Russia, Arasappan et al., 2010; Isakov et al., 2016) with earlier SARS-CoV-1 M^pro^ inhibitors containing the widely used P1 γ-lactam and electrophilic arylketone or nitrile functionalities as warheads (Thanigaimalai et al., 2013; Konno et al., 2013; Chuck et al., 2013). Our design logic also considered the observations and conclusions from our recent neutron structure of M^pro^ in complex with another hepatitis C virus protease inhibitor, telaprevir (Kneller et al., 2021c). In the M^pro^/telaprevir complex, we found the oxygen of the hemithioketal formed by Cys145 reacting with the ketone warhead is protonated and makes a distorted, possibly weak, hydrogen bond with His41. Based on this observation, we hypothesized that an inhibitor’s affinity might be increased by directing its warhead to form hydrogen bonding interactions with the oxyanion hole instead of His41.

Here, we present the design and characterization of three hybrid reversible covalent M^pro^ inhibitors named BBH-1, BBH-2 and NBH-2 (Figure 1). To map out the atomic details of inhibitors binding to the M^pro^ active site, we determined a 2.2 Å joint X-ray/neutron (XN) structure of M^pro^/BBH-1 complex and 1.8 Å X-ray structures of M^pro^/BBH-2 and M^pro^/NBH-2 complexes at near-physiological temperature and pH. For direct comparison, we also obtained a 2.0 Å room-temperature X-ray structure of M^pro^ in complex with PF-07321332. The M^pro^/BBH-1 XN structure captured changes of the protonation states relative to the inhibitor-free M^pro^ (Kneller 2020b) that could not be predicted *a priori*; in particular, the protonation states of four His residues located in the active site were directly visualized, revealing ionization states of His41, His163, His164 and His172. The XN structure also demonstrated that the hemithioketal generated by covalent binding of BBH-1 to Cys145 is not protonated, its oxygen being observed as an alkoxy anion. Inhibitors BBH-2 and NBH-2 effectively inhibited M^pro^ *in vitro*, with the dissociation constants (*K*_d_) of 26–30 nM measured by isothermal titration calorimetry (ITC), which compare favorably with the 7 nM *K*_d_ for PF-07321332 (*K*_i_=3.1 nM measured by Owen et al., 2021). Our study provides unique information on the structural and thermodynamic details of the hybrid reversible covalent inhibitors binding to SARS-CoV-2 M^pro^ and demonstrates that these compounds are viable for further design of specific antivirals against SARS-CoV-2.

## Results

### Design of hybrid M^pro^ inhibitors

We initiated design of hybrid inhibitors by careful examination of the chemical structures of boceprevir, narlaprevir and telaprevir and their mode of binding to M^pro^ based on our recent room-temperature X-ray structures (Kneller et al., 2020a). These inhibitors belong to the class of reversible covalent inhibitors, each containing an electrophilic ketone warhead that Cys145 attacks to generate the covalent hemithioketal adduct. The inhibitors chemical architectures are peptidomimetic, harboring mainly hydrophobic groups at positions P4-P1 (P5-P1 for narlaprevir, Figure 1). Importantly, X-ray crystallography provided evidence that these compounds have stereochemistry promoting their binding to M^pro^ (Kneller et al., 2020a; Ma et al., 2020; Fu et al., 2020). We have also recently shown by neutron crystallography that in the M^pro^/telaprevir complex, the hemithioketal is protonated making a distorted, possibly weak, hydrogen bond with neutral His41. We hypothesized that directing the warhead into the oxyanion hole might benefit an inhibitor’s binding affinity. We accomplished this by a) substituting the ketoamide group of boceprevir with the keto-benzothiazole moiety where the aromatic substituent is P1, or b) introducing the nitrile warhead that fully eliminates the influence of a P1 group. Next, we substituted the hydrophobic P1 groups of boceprevir and narlaprevir with a γ-lactam, an established mimic of P1 Gln, found in GC-376 and earlier inhibitors of SARS-CoV-1 M^pro^ (Thanigaimalai et al., 2013; Konno et al., 2013; Fu et al., 2020). A P1 group must have a hydrogen bonding acceptor capability to form a strong hydrogen bond with His163 in the S1 substrate binding subsite. Recent neutron crystallographic structures of M^pro^ revealed that His163 adapts from the Nδ1-D tautomer to the protonated and positively charged state in inhibitor complexes (Kneller et al., 2021b, c). We therefore designed and synthesized the hybrid reversible covalent inhibitors BBH-1, BBH-2 and NBH-2 (Figures 1 and S1). We rescinded consideration of a narlaprevir-derived inhibitor with the keto-benzothiazole moiety due to the poor solubility of BBH-1.

### Adaptation of M^pro^ active site electrostatics upon BBH-1 binding

To gain insight into the atomic details of a hybrid inhibitor binding to M^pro^, we obtained a 2.2 Å neutron crystallographic structure of the M^pro^/BBH-1 complex jointly refined with 1.85 Å X-ray diffraction data. Both datasets were collected at room temperature and neutral pH from the same deuterated protein crystal (Table S1). The joint XN structure revealed accurate locations of hydrogen atoms (observed as deuterium atoms, D), directly mapping the effect of the inhibitor binding on the active site electrostatics. For clarity, the hydrogen bonds observed in the XN structure are described by measuring distances between a D atom from a hydrogen bond donor and a heavy atom belonging to a hydrogen bond acceptor.

**Figure 2:**
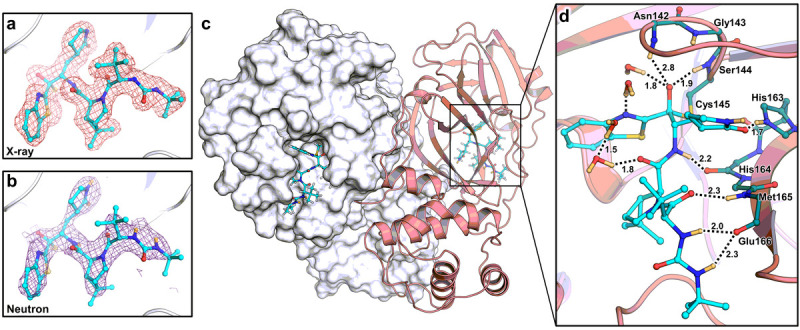
Joint X-ray/neutron crystal structure of deuterated SARS-CoV-2 M^pro^ with BBH-1. a) Electron and b) nuclear density for BBH-1 c) covalently bound in the M^pro^ active site. d) Direct and water-mediated hydrogen bond interactions between BBH-1 and M^pro^. Protein homodimer represented with one protomer as white surface and the other as salmon cartoon. Electron (pink mesh) and nuclear (purple mesh) density of BBH-1 (cyan ball and sticks) contoured to 1 σ. Hydrogen bonds are represented as black dashes with distances in angstroms (Å). Deuterium atoms are colored in light orange.

**Figure 3:**
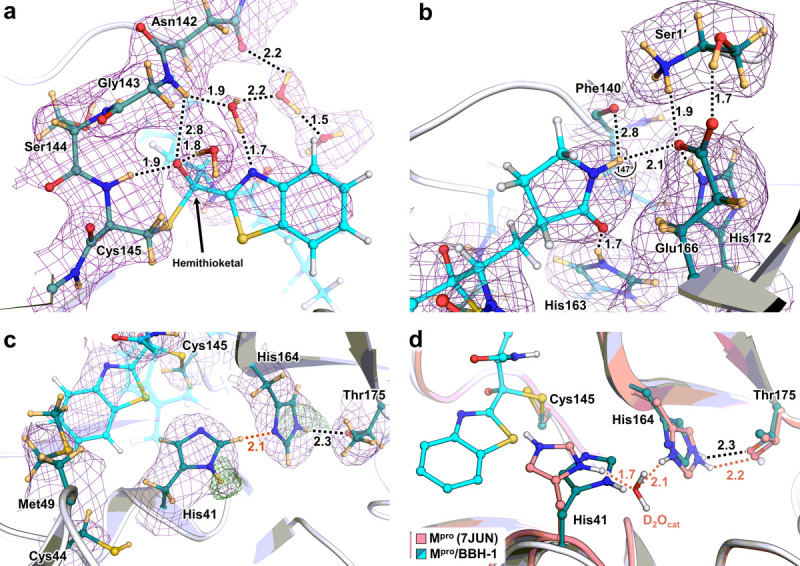
Direct observation of BBH-1 electrostatic interactions with M^pro^ determined by neutron crystallography a) Cys145 reacts with the keto-benzothiazole warhead of BBH-1 to form a covalent hemithioketal group with an alkoxy anion directed towards the oxyanion hole and hydrogen bonding to an accompanied D_2_O molecule. b) Hydrogen bond network involved with the P1 g-lactam of BBH-1 in the M^pro^ S1 pocket. c) The catalytic His41 in M^pro^/BBH-1 is singly protonated on Nδ1 and forms a C-D· · ·N interaction (orange dashes) with His164. d) The P1′ benzothiazole group of BBH-1 sterically swings His41 from its position in the ligand-free XN structure of M^pro^ (7JUN) and evicts the catalytic water molecule. Protonation states of His41 and His164 change from doubly protonated to singly protonated. BBH-1 as cyan ball and sticks. Ligand-free M^pro^ colored in salmon. Neutron 2Fo-Fc map (purple mesh) contoured at 1.0 σ. Omit nuclear Fo-Fc for D atoms on His41 and His164 are shown as green mesh contoured at 4 and 2.8 σ respectively. Hydrogen bonds represented as black dashes. Distance are given in angstroms (Å). Deuterium atoms are colored in light orange.

BBH-1 is present in both electron and nuclear density maps (Figure 2). The maps provide evidence that the Cys145 thiolate has reacted with the keto-warhead of BBH-1 to form a reversible covalent hemithioketal. According to the nuclear density (Figure 3A), the hemithioketal oxygen is not protonated and thus is observed as an alkoxy anion (C–O^−^). Its negatively charged oxygen is inserted into the oxyanion hole, and the charge is stabilized by a 1.9 Å hydrogen bond with the main-chain amide N-D of Cys145. The alkoxy anion is hydrated by a water molecule, which makes a 1.8 Å hydrogen bond with the charged oxygen. This water’s lone electron pairs interact with the P1 benzothiazole conjugated π-system through n-π* contacts with distances of 2.8–3.5 Å (Singh et al., 2016; Newberry & Raines, 2017). The benzothiazole nitrogen accepts a 1.7 Å hydrogen bond from another hydration water molecule that is also hydrogen bonded to the Gly143 main-chain N-D with a distance of 1.9 Å. The latter water molecule is part of the water network that connects benzothiazole to the Asn142 side chain and a carbonyl group of BBH-1 (Figure 2 and 3A).

The γ-lactam of the P1 group inserts into the substrate binding subsite S1 cavity formed by a stretch of residues from Phe140 to Asn142 on one side, and Glu166 and His172 on the other side (Figure 3B). The S1 subsite is further capped by Ser1 from the second M^pro^ protomer, and P1 is secured by His163 at the bottom. The γ-lactam 5-membered ring forms a conventional envelope conformation. The carbonyl oxygen makes a short 1.7 Å hydrogen bond with the protonated imidazolium side chain of His163. A similar hydrogen bond was observed between the P1 uracil of a noncovalent inhibitor Mcule-5948770040 and His163 (Kneller et al., 2021b). However, unlike the uracil group, the γ-lactam doesn’t make a C-H…O interaction with His172. Although the γ-lactam’s amide N-D is within hydrogen bonding distance (2.1 Å) to the Glu166 carboxylate, the geometry of the contact is significantly distorted from the ideal hydrogen bond geometry. The N-D…O angle is 147°, and the N-D bond vector is almost perpendicular to the carboxylate plane. Moreover, the Glu166 side chain is already hydrogen bonded with His172 (2.2 Å) and the N-terminal ND_3_^+^ of Ser1 (1.9 Å). This suggests that the γ-lactam…Glu166 contact is probably weak. Nonetheless, the Glu166 carboxylate rotates by about 90° from its observed position in the inhibitor-free M^pro^ structure. It forms a new hydrogen bond with the neutral His172, as was observed in our other inhibitor-bound M^pro^ structures (Kneller et al., 2020, 2021b, c). P2, P3 and P4 groups of BBH-1 are hydrophobic and do not form hydrogen bonds with M^pro^. Conversely, the amide groups connecting P1/P2 and P2/P3 and the substituted urea moiety make several hydrogen bonds of 2.0–2.3 Å with the main chains of His164 and Glu166. In addition, the carbonyl of the urea moiety forms an unconventional C-H… O interaction with the side chain of Gln189.

The P1 benzothiazole is located in the substrate binding subsite S1 flanked by Thr25, His41, Cys44 and Met49 (Figure 3C). Because of its steric bulkiness, the benzothiazole pushes His41 away from its position in the inhibitor-free M^pro^ structure (Kneller et al., 2020b), forcing the His41 side chain to rotate ~56° around the Cα-Cβ bond (Figure 3D). The His41 movement causes the catalytic water molecule (D_2_O_cat_) to be ejected from the active site and His164 to become deprotonated at the Nδ1 atom. A similar cascade of structural changes was previously detected in the X-ray structures of other inhibitors containing the keto-benzothiazole moiety (Hattori et al., 2021; Owen et al. 2021). Interestingly, the His41 side chain imidazole does not flip 180° in the present structure and only shifts ~0.4 Å at Cα to accommodate the benzothiazole substituent, which creates an unconventional 2.1 Å C-H…N interaction with His164 (Figure 3C). In the published X-ray structures (Hattori et al., 2021; Owen et al. 2021), the His41 imidazole side chain was modeled in the flipped conformation, suggesting the formation of an N-H…N hydrogen bond with His164 (Figure S2). In both M^pro^/BBH-1 and inhibitor-free M^pro^ XN structures, Nε2 of His164 donates its D in a hydrogen bond with the Thr175 hydroxyl, which forms a hydrogen bond with the main-chain carbonyl of Asp176. This hydrogen bond is conserved in all XN structures we have determined (Kneller et al., 2020b, 2021b, c). The neutral protonation state of His172 is also maintained because it is secured by the hydrogen bond with main-chain amide N of Gly138. Nδ1 lock Moreover, by altering the protonation states of His residues, the M^pro^ active site electrostatics adapts to the binding of BBH-1. Still, it maintains the overall charge of +1, as seen in the inhibitor-free M^pro^ (Kneller et al., 2020b).

### Binding of inhibitors containing nitrile warhead

To generate a detailed structural picture of the nitrile warhead-containing hybrid inhibitors BBH-2 and NBH-2 binding to M^pro^, we performed room-temperature X-ray crystallographic analysis of M^pro^/BBH-2 and M^pro^/NBH-2 complexes at 1.8 Å resolution. For direct comparison, we also determined a room-temperature X-ray structure of M^pro^ in complex with PF-07321332 to a resolution of 2.0 Å. The hydrogen bond distances in the X-ray structures are given below with the distances measured between the heavy atoms because, unlike in the M^pro^/BBH-1 XN structure, hydrogen atoms are not observed in the electron density at such resolutions.

**Figure 4:**
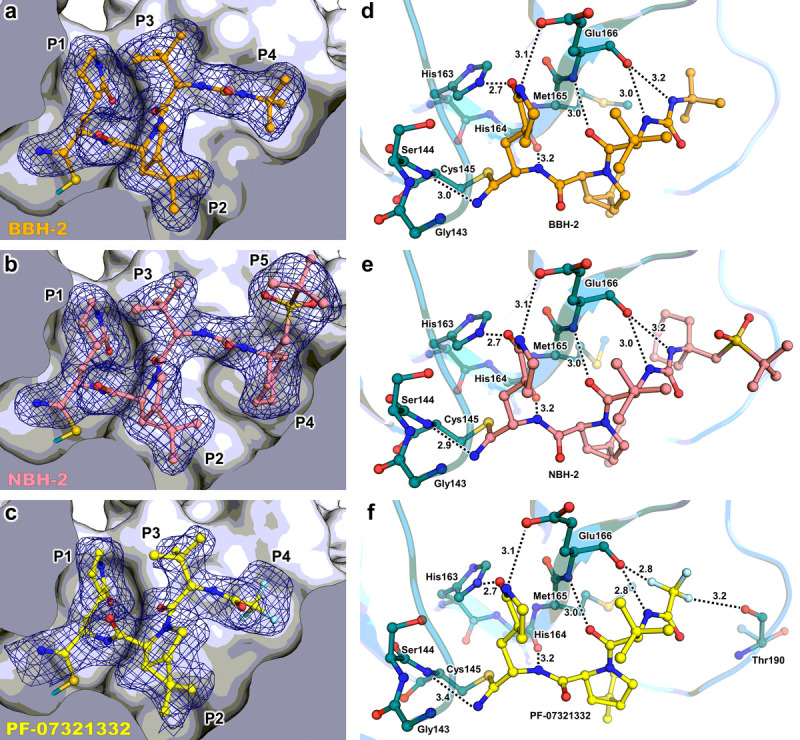
Room-temperature X-ray crystal structures of M^pro^ in complex with nitrile-warhead inhibitors BBH-2, NBH-2, and PF-037321332 a-c) Inhibitors BBH-2, NBH-2, and PF-037321332 bind to the active site of M^pro^ and form a covalent thioimidate with Cys145. Polder omit maps of inhibitor atoms (blue mesh) contoured to 3σ. d-f) Hydrogen bonds between inhibitors and M^pro^ are shown as black dashes with distances in angstroms (Å).

Both BBH-2 and NBH-2 bind in an essentially identical fashion to the M^pro^ active site. The nitrile warhead forms the covalent thioimidate conjugate with the catalytic Cys145 and is inserted into the oxyanion hole (Figure 4). The thioimidate N forms a 3.0 Å hydrogen bond with the main-chain amide NH of Cys145 but is too far from Gly143 to form a hydrogen bond (N…N distance is > 3.6 Å) and faces the Ser144 main chain almost perpendicularly. In addition, the thioimidate N is hydrated by a water molecule. The interactions are likely similar to those found for the hemithioketal alkoxy anion of BBH-1. It, therefore, appears that in the case of these inhibitors only the main chain of Cys145, and not of Gly143 or Ser144, is involved in hydrogen bonding with the covalently modified warhead. Whether the thioimidate N is protonated/neutral or deprotonated/negatively charged as observed for BBH-1 hemithioketal cannot be discerned from the X-ray structure. As observed in the M^pro^/BBH-1 XN structure, BBH-2 and NBH-2 make several hydrogen bonds with the main chain atoms of His164 and Glu166 with the distances of 3.0–3.2 Å, and a 2.7 Å hydrogen bond with the side chain of His163. Of note, the thioimidate N of PF-07321332 is farther away from Cys145 main-chain amide N at 3.4 Å, but its P1 group and the amide connecting P3 and P4 form slightly shorter hydrogen bonds of 2.6 and 2.8 Å with His163 and Glu166, respectively, compared to those seen for BBH-2 or NBH-2. Other interactions made by BBH-2, NBH-2 and PF-07321332 with M^pro^ are hydrophobic except those made by the P4 CF_3_ group of PF-07321332. The CF_3_ substituent makes 2.8 and 3.2 Å contacts with the main-chain carbonyl oxygens of Glu166 and Thr190. Such F…O interactions have previously been characterized in small-molecule compounds as attractive multipolar interactions (Xi et al., 2018).

**Figure 5:**
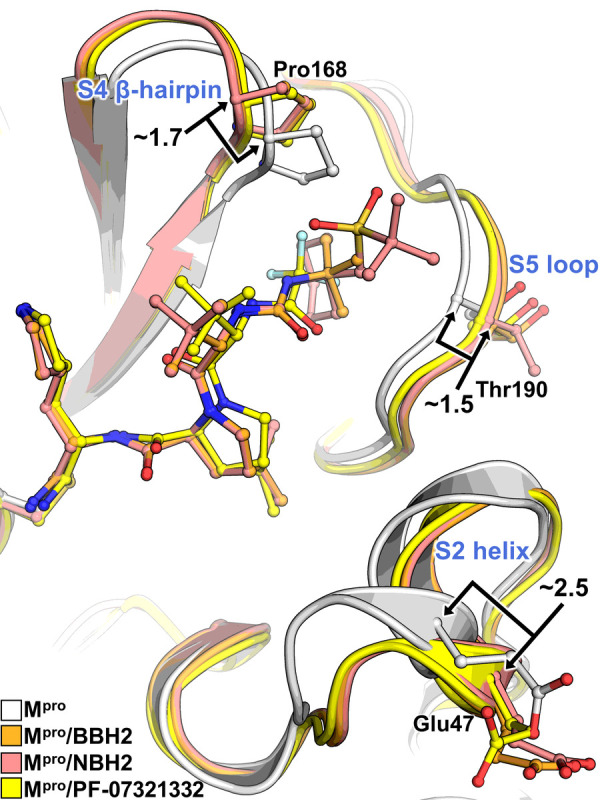
Active site structural plasticity of M^pro^ bound to nitrile-war head inhibitors Comparison of ligand-free M^pro^ (7JUN, white cartoon) with the BBH-2, NBH-2, and PF-037321332 shows significant structural shifts in protein subsites S2, S4, and S5 responsible for binding to inhibitor P2, P4, and P5 moieties respectively. Distances are given in angstroms (Å). Superposition is calculated by the least-square-fitting of Cα atoms.

We have previously demonstrated (Kneller et al., 2020a, 2020c) that the active site cavity of M^pro^ exhibits a significant degree of conformational plasticity and malleability, which assists in the binding of sterically bulky inhibitors. Specifically, the most malleable regions of the active site cavity are the S2 helix (residues 46–50), S4 β-hairpin loop (residues 165–170) and S5 loop (residues 189–194). These regions can shift by as much as 2.5 Å relative to their positions in the inhibitor-free structure (Kneller et al., 2020a). It is, therefore, instructive to compare the shapes active site cavities adopt in the M^pro^ complexes reported here with that in the inhibitor-free enzyme. Accordingly, we superimposed the four studied complexes over the XN structure of inhibitor-free M^pro^ (Kneller 2020b). Although the overall root-mean-square deviations (RMSDs) are low, ~ 0.4 Å for all structures, significant changes in the conformations of the regions directly interacting with the inhibitors are apparent (Figure 5). The S2 helix and S4 β-hairpin loop shift by as much as 2 Å, whereas the S5 loop moves by about 1.7 Å in M^pro^/BBH-1 to support induced fit of the inhibitor. The conformational changes are less drastic in M^pro^/BBH-2 and M^pro^/NBH-2 for the S4 β-hairpin loop (~ 1.7 Å) and S5 loop (~ 1.5 Å) but are more pronounced for the S2 helix (~2.5 Å), although P2 groups are identical in these inhibitors and the P4-P5 groups of NBH-2 are sterically substantially bulkier than the *tert*-butyl substituent of BBH-1 or BBH-2. In this regard, a noteworthy observation is that in the M^pro^/PF-07321332 complex, the conformational changes of the S2 helix and S4 β-hairpin loop are very similar to those seen in the other two nitrile warhead-containing inhibitors. This finding is expected for the S2 helix because the P2 groups of the inhibitors are identical. However, the P4 CF_3_ group of PF-07321332 is sizably smaller than the *tert*-butyl moiety of BBH-2 or the cyclohexyl group of NBH-2. The S4 β-hairpin loop shift may partially be caused by electrostatic repulsion with the highly electronegative CF_3_ group. Conversely, the S5 loop moves by just ~ 1 Å in M^pro^/PF-07321332 relative to the inhibitor-free M^pro^ in agreement with the reduced steric size of the CF_3_ group and the lack of a P5 group present in NBH-2. Overall, the above structural analysis reinforces the idea that the M^pro^ active site cavity can accommodate various chemical groups by induced fit, especially in the subsites S4 and S5. The room-temperature structure of the M^pro^/PF-07321332 complex superimposes onto the 100K structure of this complex reported previously (Owen et al. 2021) with an RMSD of 0.5 Å (Figure S3). It appears, however, that the M^pro^ active site is more open at room temperature. The distances between the S2 helix and the S4 β-hairpin loop, and between the S5 loop and the S4 β-hairpin loop increase by ~0.7 Å in the room-temperature structure relative to those in the 100 K structure. This could be due to differences in packing interactions between the unit cell types and the different data collection temperatures, but the change underscores the very flexible nature of the enzyme’s active site.

### Binding thermodynamics of the inhibitors

To determine the dissociation constants (*K*_d_) and assess the binding thermodynamics of nitrile warhead-containing inhibitors BBH-2, NBH-2 and PF-07321332, we performed isothermal titration calorimetry (ITC, Table 1 and Figure S4). We could not carry out ITC measurements for BBH-1 due to insufficient solubility of the inhibitor in buffer at the concentrations required for ITC even in the presence of 1.5% DMSO. BBH-2 and NBH-2 demonstrated high binding affinity to M^pro^, with almost identical *K*_d_ values of 0.026±0.016 and 0.030±0.007 μM. Moreover, PF-07321332 binds to M^pro^ with several fold lower *K*_d_, demonstrating its superior binding affinity compared to the other two inhibitors and in agreement with the *K*_i_ of 0.0031 μM reported previously (Owen et al., 2021). Binding of these inhibitors to M^pro^ is driven largely by enthalpy. Notably, ΔH and ΔS of binding for BBH-2 and NBH-2 are virtually identical, even though their P4 groups are different, and BBH-2 lacks a P5 group. This indicates that the NBH-2’s P5 group has no measurable effect on its binding to M^pro^. The ΔH of binding for PF-07321332 is more favorable than those for BBH-2 and NBH-2 by about −2 kcal/mol, implying that P4 CF_3_ group makes better interactions with M^pro^ amino acid residues compared to the hydrophobic groups on the other two inhibitors. The improvement in ΔH of binding for PF-07321332, however, is somewhat compensated by the less favorable ΔS of binding relative to BBH-2 and NBH-2. The difference in the ΔS of binding could be due to greater entropy of dehydration, which is required for the bulky hydrophobic P4 and P5 groups in BBH-2 and NBH-2 to bind to M^pro^ compared to that for the small CF_3_ group present in PF-07321332. Nevertheless, PF-07321332 binds to the enzyme with a very small change in entropy, akin to the binding of the HL-3 series of noncovalent inhibitors (Kneller et al., 2021b). Consequently, although PF-07321332 possesses a higher binding affinity than those of BBH-2 and NBH-2, the interplay of ΔH and ΔS components manifests in the free energy of binding (ΔG) for PF-07321332 being only −0.7 kcal/mol more favorable than ΔG of binding for the other two inhibitors.

### Antiviral activity

The *in vitro* antiviral activity of all four compounds, BBH-1, BBH-2, NBH-2, and PF-07321332, was evaluated in Vero E6 TMRSSS cells. These cells express significant amounts of P-glycoprotein that acts as an efflux pump capable of efficiently removing these compounds from the cytoplasm (Owen et al., 2021; Boras et al., 2021). Therefore, we assessed the antiviral properties of the inhibitors in the absence and presence of CP-100356, a P-glycoprotein inhibitor. In the absence of CP-100356, BBH-1, BBH-2 and NBH-2 inhibited viral replication rather weakly, with EC_50_s in the 14–16 μM range, whereas PF-07321332 showed a more potent EC_50_ of 0.88 μM (Table 2, Figure S5). Addition of 2 μM CP-100367 significantly improved the antiviral potency of all the compounds, with the EC_50_ values decreasing at least 10-fold for BBH-1 (1.5 μM), BBH-2 (0.88 μM) and NBH-2 (1.82 μM). In the presence of CP-100356, PF-07321332 inhibited SARS-CoV-2 replication with EC_50_ of 0.25 μM, which is consistent with the recently reported measurements (Owen et el. 2021). No cytotoxicity was detected for all four inhibitors at concentrations up to 10 μM.

## Discussion

The current design approach for specific small-molecule inhibitors of SARS-CoV-2 M^pro^ has yielded an orally bioavailable compound PF-07321332 (Nirmatrelvir, Paxlovid™, Pfizer Inc.), a reversible covalent peptidomimetic inhibitor that takes advantage of the nucleophilic properties of the catalytic Cys145 thiolate and whose chemical structure combines features of boceprevir, GC-376 and other earlier inhibitors of SARS-CoV-1 M^pro^. Further design of novel M^pro^ inhibitors, including covalent and noncovalent compounds, will undoubtedly be required to battle COVID-19 and to prepare for the possibility of future coronavirus outbreaks. Improved inhibitor design should consider employing both conventional hydrogen bonds and unconventional intermolecular interactions, such as C-H···O(N) and X-H···π contacts, where X = O, N, S, or C (McConnell, 2021), with M^pro^ residues. Thus, hydrogen atoms that represent about half of the total atom count in both small molecules and proteins cannot be overlooked. We have been using neutron protein crystallography to create a map of hydrogen atom positions and their movements that alter active site electrostatics due to inhibitor binding in SARS-CoV-2 M^pro^ (Kneller et al., 2020b, 2021b, c). This led us to the design of hybrid covalent inhibitors BBH-1, BBH-2 and NBH-2 with a 100-fold improved affinity to M^pro^ compared to that of boceprevir and narlaprevir (Ma et al., 2020; Fu et al., 2020; Kneller et al. 2020a), whose chemical structures were the starting points in our design approach. These inhibitors together with PF-07321332 also exhibited robust antiviral properties, inhibiting SARS-CoV-2 replication in our assays at the low-to-sub μM concentrations.

The most important observations in the XN structure of M^pro^/BBH-1 are the protonation states and hydrogen bonding contacts visualized in the oxyanion hole and S1 substrate binding subsite. As expected from the mechanism of cysteine protease-catalyzed peptide bond hydrolysis, the hemithioketal oxygen of BBH-1 was found not to be protonated. Its negative electric charge is stabilized by a hydrogen bond with the main chain amide N-H of Cys145 (Figure 3). The P1 γ-lactam makes a strong hydrogen bond with His163 that becomes protonated and positively charged when BBH-1 binds to M^pro^. This hydrogen bond is essential for inhibitor binding affinity, as its disruption leads to a significant loss in inhibitor potency, as was shown in our structure-activity relationship study on a noncovalent inhibitor (Kneller et al., 2021b). Similar protonation states and hydrogen bond interactions can be envisaged in the M^pro^ complexes with the three nitrile warhead inhibitors studied here, in which the thioimidate nitrogen would be negatively charged. Protonation of His163 to gain a positive charge upon binding of an inhibitor irrespective of the P1 group’s chemical nature is evidently an intrinsic property of this residue’s side chain, a conclusion drawn based on our observations of this phenomenon in all neutron structures that we have determined so far (Kneller et al., 2020b, 2021b, c). Moreover, another remarkable property of the M^pro^ active site that appears to be invariant is its ability to maintain overall +1 electric charge when inhibitors bind through switching protonation states of His41, Cys145, His163 and His164 (Table S3).

Binding of the nitrile warhead-containing inhibitors BBH-2, NBH-2 and PF-07321332 is enthalpically driven, an important property for a successful drug molecule. Although the three inhibitors are similar, the entropy of binding for the two former inhibitors is significant, whereas it is small for PF-07321332 and is substantially compensated by a more favorable enthalpy of binding. This makes PF-07321332 a superior inhibitor among the three compounds studied with ITC. This result is probably caused by substituting the bulky hydrophobic P4/P5 groups of BBH-2 and NBH-2 with a small electronegative CF_3_ group that minimizes the hydrophobic effect and is also capable of forming favorable unconventional F···O interactions with M^pro^. Thus, the CF_3_ group conveys more favorable antiviral properties (Table 2) and was also shown to be beneficial for the compound’s metabolic stability and pharmacokinetics (Owen et al., 2021). Future M^pro^ inhibitor design can consider altering the P3 *tert*-Bu group that is exposed to solvent and makes no contacts with M^pro^ residues.

## Conclusion

In summary, we designed three hybrid reversible covalent inhibitors of SARS-CoV-2 M^pro^ based on the chemical structures of hepatitis C clinical protease inhibitors narlaprevir and boceprevir and compared them to the recently FDA-approved inhibitor PF-07321332. Our study combined chemical synthesis, neutron and X-ray crystallography at nearly physiological temperature, and *in vitro* measurements to obtain atomic details of the inhibitors’ binding to M^pro^, their binding thermodynamics and antiviral potency. For the first time, we directly visualized an oxyanion bound in the catalytic site of M^pro^, the negatively charged oxygen being generated when Cys145 reacts with the ketone warhead of the BBH-1 inhibitor. BBH-1, BBH-2 and NBH-2 showed significant antiviral activity that was only slightly lower than that for PF-07221332. Our results shed new light on the atomic details of inhibitor binding to SARS-CoV-2 M^pro^ and provide insights for future drug design.

## Materials And Methods

### General Information

Ni-NTA columns were purchased from Cytiva (Piscataway, New Jersey, USA). His-tagged Human Rhinovirus (HRV) 3C protease was purchased from Sigma (MilliporeSigma, St. Louis, MO). Crystallization reagents and supplies were purchased from Hampton Research (Aliso Viejo, California, USA). Crystallographic supplies for crystal mounting and X-ray and neutron diffraction data collection at room temperature were purchased from MiTeGen (Ithaca, New York, USA) and Vitrocom (Mountain Lakes, New Jersey, USA). PF-07321332 (CAS # 2628280-40-8) was purchased from MedChemExpress (Monmouth Junction, New Jersey, USA)

### Synthesis of BBH-1, BBH-2, and NBH-2

Boceprevir and narlaprevir both possess alkyl substituted 3-amino-2-oxopropanamide endgroups. It was our intent to replace those moieties with P1 fragments containing the g-lactam group and the desired nitrile or keto-benzothiazole warhead. To prepare the boceprevir-derived hybrids BBH-1 and BBH-2, the boceprevir fragment lacking the 3-amino-4-cyclobutyl-2-oxobutanamide moiety was utilized. This fragment, (1*R*,2*S*,5*S*)-3-((*S*)-2-(3-(*tert*-butyl)ureido)-3,3-dimethylbutanoyl)-6,6-dimethyl-3-azabicyclo[3.1.0]hexane-2-carboxylic acid, was coupled to either (*S*)-3-((*S*)-2-amino-3-(benzo[*d*]thiazol-2-yl)-3-oxopropyl)pyrrolidin-2-one (for BBH-1), or (*S*)-2-amino-3-((*S*)-2-oxopyrrolidin-3-yl)propanenitrile (for BBH-2), using standard amide forming methodologies employed in peptide coupling reactions. Similarly, to prepare NBH-2, the narlaprevir fragment (1*R*,2*S*,5*S*)-3-((*S*)-2-(3-(1-((*tert*-butylsulfonyl)methyl)cyclohexyl)ureido)-3,3-dimethylbutanoyl)-6,6-dimethyl-3-azabicyclo[3.1.0]hexane-2-carboxylic acid was coupled with (*S*)-2-amino-3-((*S*)-2-oxopyrrolidin-3-yl)propanenitrile (see Figure S1). Details of the syntheses are described in the Supporting Information.

### Expression and purification of hydrogenated and partially deuterated M^pro^

The SARS-CoV-2 M^pro^ (NSP5) gene sequence was codon optimized before being cloned into a plasmid harboring a kanamycin resistance cassette (pD451-SR, Atum, Newark, CA). The protein construct features M^pro^ flanked upstream by maltose binding protein (MBP) and downstream by a His6 tag (Kneller et al., 2020c). A native N-terminus is attained during expression through an M^pro^ autoprocessing site corresponding to the cleavage between NSP4 and NSP5 in the viral polyprotein, SAVLQ↓SGFRK, where ↓ denotes the cleavage site. A native C-terminus is produced through an HRV-3C protease cleavage site (SGVTFQ↓GP). Hydrogenated M^pro^ was expressed in *Escherichia coli* and purified according to the established procedures (Kneller et al., 2020c, d). Partially deuterated M^pro^ was expressed using a bioreactor and purified as described recently (Kneller et al., 2021c). Protein yields for hydrogenated M^pro^ preparations averaged ~3.5 mg per 1 g cells and were used fresh or stored at −30°C. Final yield for the partially deuterated purification was ~1.6 mg per 1 g of cell paste and was used immediately for crystallization. Similar to the above strategy, a second construct was used to express and purify the wild-type M^pro^. Expression and purification were carried out as described previously (Kneller et al., 2021b and Zhang et al., 2020). This second source of M^pro^ was used to determine the binding constants by ITC.

### M^pro^-inhibitor complex crystallization

Instructions for reproducing large-volume plate-shaped crystals of hydrogenated and partially deuterated M^pro^ enzyme are accessible (Kneller et al., 2020d). Discovery of conditions for crystallizing M^pro^ was accelerated by automated high-throughput screening at the Hauptman-Woodward Medical Research Institute (HWI) (Luft et al., 2003). Crystal aggregates of ligand-free M^pro^ were reproduced locally then converted into microseeds for growing single crystals. We recently discovered that microseeded M^pro^ crystals grow exclusively in a cuboid morphology when co-crystallized with noncovalent ligand Mcule-5948770040 and its derivatives (Kneller et al., 2021b). Cube-shaped crystals of M^pro^ for current experiments were obtained by seed streaking from cube-shaped M^pro^/Mcule-5948770040 co-crystals using a cat whisker or seeding tool.

Hydrogenated protein in 20 mM Tris, 150 mM NaCl, and 1 mM TCEP (tris(2-carboxyethyl)phosphine) pH 8.0 was concentrated to ~5–7 mg/mL for growing ligand-free crystals and co-crystals. Stocks of inhibitors were prepared at 50 mM in 100% dimethyl sulfoxide (DMSO) for crystallization purposes and stored at −30°C. For co-crystallization, M^pro^ was mixed with BBH-1, BBH-2, or NBH-2 at 1:5 molar ratio and allowed to incubate at room temperature for a minimum of 30 minutes before setting up crystal trays. Crystals grown for room-temperature X-ray diffraction used sitting drop vapor diffusion methodology with 18–21% PEG3350, 0.1 M Bis-Tris pH 6.5 as the precipitant solution. Crystallization drops of 20 μL at 1:1 ratio were seed struck with cubes as described above. Crystals appeared after 3 days of incubation at 14°C and continued to grow to a typical volume of ~0.1 mm^3^. Co-crystallization attempts with PF-7321332 failed, prompting crystal soaking. A pre-grown ligand-free M^pro^ crystal was transferred to a different drop supplemented with PF-7321332 to a final concentration of 1.5 mM and 3% DMSO and allowed to soak for 10 minutes at room temperature before X-ray data collection. Crystals were mounted in MiTeGen (Ithaca, NY) room-temperature capillary setups for data collection.

For joint XN crystallography, partially deuterated M^pro^ at 10.6 mg/mL was mixed with BBH-1 at a 1:5 molar ratio, incubated at room temperature for 30 minutes, then filtered through a 0.2 μm centrifugal filter. A Hampton 9-well sandwich box was set up with 240 μL drops at a 1:1 ratio of protein to 20% PEG3350, 0.1 M Bis-Tris pH 6.5 reservoir solution. Due to the favorable 3D shape for neutron data collection, nucleation in the cube-morphology was encouraged by seed streaking. After 19 days of incubation at 10°C, a crystal measuring ~1.2⋅1⋅0.4 mm (0.5 mm^3^) was mounted in a quartz capillary (Figure S6). The crystal was accompanied by 22% PEG3350 prepared in 100% D_2_O to exchange labile hydrogens during the 2 weeks preceding neutron data collection. A separate crystal from the same drop was mounted inside a glass capillary in the same fashion for room-temperature X-ray data collection used in joint X-ray/neutron refinement. The final pH of the crystallization drop was measured by a microelectrode to be 6.6, corresponding to a neutral pD of 7.0 (pD = pH + 0.4). This pD is identical to previously characterized neutron crystal structures of inhibitor-free M^pro^, the M^pro^/telaprevir covalent complex, and the M^pro^/Mcule-5948770040 noncovalent complex (Kneller et al., 2020b, 2021b, c).

### Room temperature X-ray diffraction data collection and structure refinement

All room temperature X-ray crystallographic data was collected with a Rigaku HighFlux HomeLab instrument equipped with a MicroMax-007 HF X-ray generator, Osmic VariMax optics, and a DECTRIS Eiger R 4M hybrid photon counting detector. X-ray diffraction data were integrated using the CrysAlis Pro software suite (Rigaku Inc., The Woodlands, TX) then reduced and scaled using Aimless (Evans & Murshudov, 2013) from the CCP4 suite (Winn et al., 2011). Structures were solved by molecular replacement using Phaser (McCoy et al., 2007) from CCP4 using PDB code 7N8C (Kneller et al., 2021b). Each model was iteratively refined with Phenix.refine from the PHENIX suite (Adams et al., 2010; Liebschner et al., 2019) and COOT (Emsley et al., 2010; Casañal et al., 2020). Geometry validation was aided by Molprobity (Chen et al., 2010). All ligand restraints were generated with eLBOW (Moriarty et al., 2009) using geometry optimized by quantum mechanical calculations in Gaussian 16 at B3LYP/6–31g(d,p) level of theory (Frisch et al., 2016). Final data collection and refinement statistics can be found in Table S2.

### Neutron diffraction data collection

Neutron diffraction was tested at room temperature on the IMAGINE (Meilleur et al., 2020; Coates et al., 2018; Meilleur et al., 2013, 2018) instrument located at the High Flux Isotope Reactor (Oak Ridge National Laboratory) using the broad bandpass functionality with neutron wavelengths between 2.8 and 10 Å. Neutron quasi-Laue diffraction data from a 0.5 mm^3^ crystal of the M^pro^/BBH-1 complex were collected at room temperature using the LADI-III diffractometer (Blakeley et al., 2010) at the Institut Laue-Langevin (ILL) in Grenoble. A neutron wavelength range (Δλ/λ = 30%) of 2.80–3.75 Å was used for data collection with diffraction data extending to 2.2 Å resolution. The crystal was held stationary at different φ (vertical rotation axis) for each exposure. A total of 22 images were recorded with an average exposure time of 23 hours per image from 3 different crystal orientations. The neutron data were processed using the Daresbury Laboratory LAUE suite program *LAUEGEN* modified to account for the cylindrical geometry of the detector (Campbell, 1995; Campbell et al., 1998). The program *LSCALE* (Arzt et al., 1999) was used to determine the wavelength-normalization curve using the intensities of symmetry-equivalent reflections measured at different wavelengths. No explicit absorption corrections were applied. The data were merged and scaled using *SCALA* (Weiss, 2001). Neutron data collection can be found in Table S1.

### Joint X-ray/neutron (XN) refinement

The joint XN structure of the deuterated M^pro^/BBH-1 complex was refined using *nCNS* (Adams et al., 2009; Mustyakimov & Langan, 2007) and manual structure manipulation using COOT (Emsley et al., 2010; Casañal et al., 2020). Initial rigid-body refinement was followed by several cycles of positional, atomic displacement parameter, and occupancy refinement. Correctness of sidechain conformations, hydrogen bonding, and orientations of D_2_O water molecules in the structure was determined from *mF*_O_-*DF*_C_ difference neutron scattering length density maps. The 2*mF*_O_-*DF*_C_ and *mF*_O_-*DF*_C_ neutron scattering length density maps were then examined to determine the correct orientations of hydroxyl (Ser, Thr, Tyr), thiol (Cys), and ammonium (Lys) groups, and protonation states of the enzyme residues. The protonation states of some disordered side chains on the protein surface could not be obtained directly and remained ambiguous. Water molecules were refined as D_2_O. Water oxygen atoms were centered on their electron density peaks, and each molecule was rotated in accordance with the neutron scattering length density maps. Hydrogen positions in the protein were modeled as deuterium atoms because the protein was partially deuterated. Occupancies of D atoms were refined individually within the range of −0.56 (pure H) to 1.00 (pure D) because the neutron scattering length of H is −0.56 times that of D. Before depositing the neutron structure to the PDB, coordinates of a D atom were split into two records corresponding to an H and a D partially occupying the same site, both with positive partial occupancies that add up to unity. The percent D at a specific site is calculated according to the following formula: % D = {occupancy(D) + 0.56}/1.56. Neutron refinement statistics can be found in Table S1.

### Isothermal Titration Calorimetry

Purified M^pro^ was diluted from a stock solution to 60 μM and dialyzed overnight at 4°C against 25 mM Tris-HCl, pH 7.2, 20 mM NaCl and 1 mM TCEP (ITC buffer). The concentration of M^pro^ was estimated based on its 280 nm absorbance. Stock solutions of inhibitors dissolved in DMSO were freshly diluted in ITC buffer prior to the titration and contained a final concentration not exceeding 1.5% DMSO. The protein solution was also adjusted to contain the same concentration of DMSO in ITC buffer. Titrations were performed with 30 μM M^pro^ in the cell and ~10-fold molar excess of titrant at 28°C on an iTC200 microcalorimeter (Malvern Instruments Inc., Westborough, MA). Control titrations of buffer with each of the inhibitors showed a negligible response. Data were processed and plots were generated using the Origin software provided with the instrument. For competitive inhibitors that bind at only one site, the dissociation constant (*K*_d_ = 1/*K*_a_) is equivalent to the inhibition constant measured by enzyme kinetics (*K*_i_).

### Antiviral assays

Evaluation of antiviral activity of compounds BBH-1, BBH-2, NBH-2 and PF-7321332 was carried out in a 384-well plate with Vero E6 TMPRSSS cells as described in Bocci et al. (2020) using the USA-WA1/2020 (deposited by the Centers for Disease Control and Prevention and obtained through BEI Resources, NIAID, NIH, NR-52281). All activities with live virus were conducted at the UTHSC Regional Biocontainment Laboratory BSL-3. Compounds were evaluated in a dose response format starting at 10 micromolar and 6 additional 2-fold dilutions in duplicate. The cytotoxicity of compounds was tested either alone or in the presence of the P-glycoprotein inhibitor CP-100356. The antiviral activity was also assessed with the compound alone or in the presence of CP-100356 and SARS-CoV-2. CP-100356 was included at 2 μM concentration to assess whether our test compounds are being effluxed out of cells due to endogenous expression of P-glycoprotein in the cells line, which is known for PF-7321332 (Owen et al. 2021). Following incubation for 48 hours at 5% CO_2_ and 37°C, the percent cell viability was measured with CellTiterGlo. Signals were read with an EnVision® 2105 multimode plate reader. Cells alone (positive control) and cells plus virus (negative control) were set to 100% and 0% cell viability to normalize the data from the compound testing. The normalized data was used to calculate the 50% cytotoxic concentration (CC_50_) and effective concentration (EC_50_) using GraphPad version 9.3.1 by plotting the log(inhibitor) vs. normalized response with a variable slope (Hill=1) (Table 2 and Figure S5).

## Supplementary Material

1

2

3

4

5

## Figures and Tables

**Table 1:** Binding affinities of nitrile warhead-containing inhibitors BBH-2, NBH-2 and PF-07321332 determined by isothermal titration calorimetry (*K*_d_ and thermodynamic parameters ΔH, ΔS and ΔG of binding).

Compound	*K*_d_, μM	Stoichiometry, N	ΔH, kcal·mol^−1^	ΔS, cal·mol^−1^·K^−1^	ΔG, kcal·mol^−1^
BBH-2	0.030 ± 0.007	1.000 ± 0.005	−8.74 ± 0.08	5.40	−10.4
NBH-2	0.026 ± 0.016	0.990 ± 0.009	−8.76 ± 0.17	5.63	−10.5
PF-07321332	0.007 ± 0.003	0.990 ± 0.003	−10.75 ± 0.70	1.57	−11.2
GC-376	0.15 ± 0.03^a^	0.99 ± 0.01	−6.7 ± 0.1	9.1	−9.4

a ITC values for GC-376 are taken from Nashed et al., 2022.

**Table 2:** In vitro antiviral parameters for SARS-CoV-2 M^pro^ inhibitors measured in Vero E6 TMPRSSS cells in the absence or presence of P-glycoprotein inhibitor CP-100356.

Compound	*EC_50_*, μM (without CP-100356)	*EC_50_*, μM (with CP-100356)	*CC_50_*, μM
BBH-1	16.1	1.5	> 10
BBH-2	15.4	0.88	> 10
NBH-2	13.9	1.82	> 10
PF-07321332	0.88	0.25	> 10

## Abbreviations

SARS-CoV-2: severe acute respiratory syndrome coronavirus 2
3CL M^pro^: chymotrypsin-like main protease
M^pro^: wild-type main protease of SARS-CoV-2
