# Supplementary material for: Covalent narlaprevir- and boceprevir-derived hybrid inhibitors of SARS-CoV-2 main protease: room-temperature X-ray and neutron crystallography, binding thermodynamics, and antiviral activity
Source: Res Sq. 2022 Feb 11:rs.3.rs-1318037. Preprint. [Version 1] doi: 10.21203/rs.3.rs-1318037/v1 (PMC8845512; doi:10.21203/rs.3.rs-1318037/v1)
Supplement: 3 [file 66addf7a9b1f2510ea58f35d.pdf]

Full wwPDB X-ray Structure Validation Report (i)

Jan 7, 2022 – 02:15 PM EST

PDB ID	:	7TDU
Title	:	Joint X-ray/neutron structure of SARS-CoV-2 main protease (3CL Mpro) in complex with BBH-1
Deposited on	:	2022-01-03
Resolution	:	1.85 Å(reported)

This is a Full wwPDB X-ray Structure Validation Report.

This report is produced by the wwPDB biocuration pipeline after annotation of the structure.

We welcome your comments at *validation@mail.wwpdb.org*
A user guide is available at
https://www.wwpdb.org/validation/2017/XrayValidationReportHelp
with specific help available everywhere you see the (i) symbol.

The following versions of software and data (see references (i)) were used in the production of this report:

MolProbity	:	4.02b-467
Mogul	:	1.8.5 (274361), CSD as541be (2020)
Xtriage (Phenix)	:	1.13
EDS	:	FAILED
buster-report	:	1.1.7 (2018)
Percentile statistics	:	20191225.v01 (using entries in the PDB archive December 25th 2019)
Ideal geometry (proteins)	:	Engh & Huber (2001)
Ideal geometry (DNA, RNA)	:	Parkinson et al. (1996)
Validation Pipeline (wwPDB-VP)	:	2.25

CONFIDENTIAL VALIDATION REPORT

1 Overall quality at a glance (i)

The following experimental techniques were used to determine the structure:
X-RAY DIFFRACTION, NEUTRON DIFFRACTION

The reported resolution of this entry is 1.85 Å.

Percentile scores (ranging between 0-100) for global validation metrics of the entry are shown in the following graphic. The table shows the number of entries on which the scores are based.

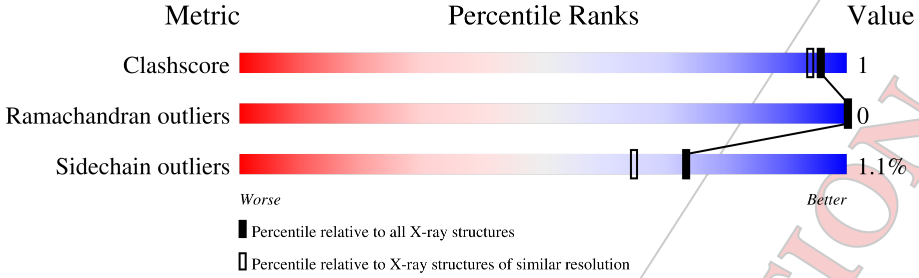

Metric	Whole archive (#Entries)	Similar resolution (#Entries, resolution range(Å))
Clashscore	141614	2625 (1.86-1.86)
Ramachandran outliers	138981	2592 (1.86-1.86)
Sidechain outliers	138945	2592 (1.86-1.86)

The table below summarises the geometric issues observed across the polymeric chains and their fit to the electron density. The red, orange, yellow and green segments of the lower bar indicate the fraction of residues that contain outliers for >=3, 2, 1 and 0 types of geometric quality criteria respectively. A grey segment represents the fraction of residues that are not modelled. The numeric value for each fraction is indicated below the corresponding segment, with a dot representing fractions <=5%

Note EDS failed to run properly.

Mol	Chain	Length	Quality of chain
1	A	306	99% •

CONFIDENTIAL VALIDATION REPORT

2 Entry composition (i)

There are 3 unique types of molecules in this entry. The entry contains 6693 atoms, of which 1572 are hydrogens and 2561 are deuteriums.

In the tables below, the ZeroOcc column contains the number of atoms modelled with zero occupancy, the AltConf column contains the number of residues with at least one atom in alternate conformation and the Trace column contains the number of residues modelled with at most 2 atoms.

- Molecule 1 is a protein called 3C-like proteinase.

Mol	Chain	Residues	Atoms							ZeroOcc	AltConf	Trace
			Total	C	D	H	N	O	S			
1	A	306	6161	1499	2263	1530	402	445	22	0	302	0

- Molecule 2 is (1R,2S,5S)-N-{(1S,2S)-1-(1,3-benzothiazol-2-yl)-1-hydroxy-3-[(3S)-2-oxo(1- 2 H)pyrrolidin-3-yl]propan-2-yl}-3-{N-[tert-butyl(2 H)carbamoyl]-3-methyl-L-(N- 2 H)val yl}-6,6-dimethyl-3-azabicyclo[3.1.0]hexane-2-(2 H)carboxamide (three-letter code: I1W) (formula: $C_{33}H_{48}N_6O_5S$) (labeled as "Ligand of Interest" by depositor).

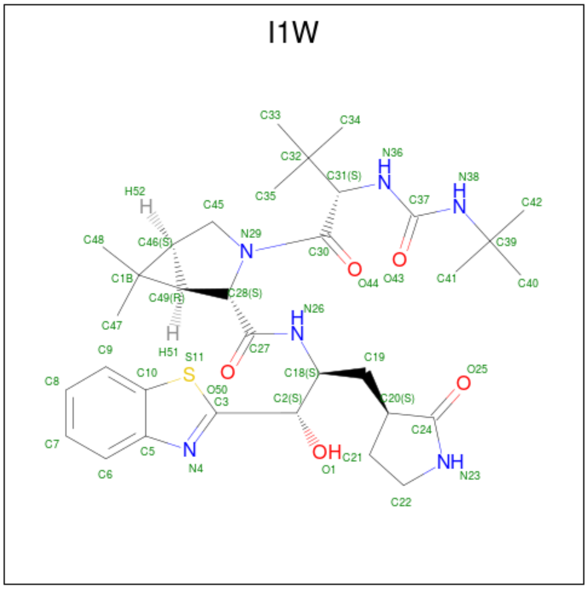

Mol	Chain	Residues	Atoms							ZeroOcc	AltConf
			Total	C	D	H	N	O	S		
2	A	1	91	33	4	42	6	5	1	0	0

- Molecule 3 is water.

Mol	Chain	Residues	Atoms			ZeroOcc	AltConf
			Total	D	O		
3	A	147	441	294	147	0	0

CONFIDENTIAL VALIDATION REPORT

3 Residue-property plots (i)

These plots are drawn for all protein, RNA, DNA and oligosaccharide chains in the entry. The first graphic for a chain summarises the proportions of the various outlier classes displayed in the second graphic. The second graphic shows the sequence view annotated by issues in geometry. Residues are color-coded according to the number of geometric quality criteria for which they contain at least one outlier: green = 0, yellow = 1, orange = 2 and red = 3 or more. Stretches of 2 or more consecutive residues without any outlier are shown as a green connector. Residues present in the sample, but not in the model, are shown in grey.

Note EDS failed to run properly.

- Molecule 1: 3C-like proteinase

Chain A:

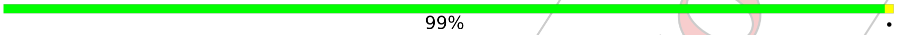

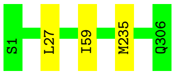

CONFIDENTIAL VALIDATION REPORT

4 Data and refinement statistics ⓘ

EDS failed to run properly - this section is therefore incomplete.

Property	Value	Source
Space group	I 1 2 1	Depositor
Cell constants<br>a, b, c, α, β, γ	55.02Å 81.22Å 88.83Å<br>90.00° 96.72° 90.00°	Depositor
Resolution (Å)	29.88 – 1.85	Depositor
% Data completeness<br>(in resolution range)	86.6 (29.88-1.85)	Depositor
R_{merge}	0.08	Depositor
R_{sym}	(Not available)	Depositor
$< I/\sigma(I) >$ [1]	2.23 (at 1.86Å)	Xtriage
Refinement program	nCNS 1.0.0	Depositor
R, R_{free}	0.197 , 0.210	Depositor
Wilson B-factor (Å^2)	27.7	Xtriage
Anisotropy	0.183	Xtriage
L-test for twinning[2]	$< \|L\| >$ = 0.50, $< L^2 >$ = 0.33	Xtriage
Estimated twinning fraction	No twinning to report.	Xtriage
Total number of atoms	6693	wwPDB-VP
Average B, all atoms (Å^2)	33.0	wwPDB-VP

Xtriage's analysis on translational NCS is as follows: *The largest off-origin peak in the Patterson function is 7.74% of the height of the origin peak. No significant pseudotranslation is detected.*

[1] Intensities estimated from amplitudes.
[2] Theoretical values of $< |L| >$, $< L^2 >$ for acentric reflections are 0.5, 0.333 respectively for untwinned datasets, and 0.375, 0.2 for perfectly twinned datasets.

CONFIDENTIAL VALIDATION REPORT

5 Model quality (i)

5.1 Standard geometry (i)

Bond lengths and bond angles in the following residue types are not validated in this section: DOD, I1W

The Z score for a bond length (or angle) is the number of standard deviations the observed value is removed from the expected value. A bond length (or angle) with $|Z| > 5$ is considered an outlier worth inspection. RMSZ is the root-mean-square of all Z scores of the bond lengths (or angles).

Mol	Chain	Bond lengths		Bond angles	
		RMSZ	#$|Z|$ >5	RMSZ	#$|Z|$ >5
1	A	0.47	0/4822	0.69	0/6552

There are no bond length outliers.

There are no bond angle outliers.

There are no chirality outliers.

There are no planarity outliers.

5.2 Too-close contacts (i)

In the following table, the Non-H and H(model) columns list the number of non-hydrogen atoms and hydrogen atoms in the chain respectively. The H(added) column lists the number of hydrogen atoms added and optimized by MolProbity. The Clashes column lists the number of clashes within the asymmetric unit, whereas Symm-Clashes lists symmetry-related clashes.

Mol	Chain	Non-H	H(model)	H(added)	Clashes	Symm-Clashes
1	A	4631	1530	15	0	0
2	A	49	42	0	2	0
3	A	441	0	0	1	2
All	All	5121	1572	15	3	2

The all-atom clashscore is defined as the number of clashes found per 1000 atoms (including hydrogen atoms). The all-atom clashscore for this structure is 1.

All (3) close contacts within the same asymmetric unit are listed below, sorted by their clash magnitude.

Atom-1	Atom-2	Interatomic distance (Å)	Clash overlap (Å)

CONFIDENTIAL VALIDATION REPORT

All (2) symmetry-related close contacts are listed below. The label for Atom-2 includes the symmetry operator and encoded unit-cell translations to be applied.

Atom-1	Atom-2	Interatomic distance (Å)	Clash overlap (Å)
3:A:548:DOD:O	3:A:587:DOD:D1[2_655]	1.49	0.71
3:A:517:DOD:O	3:A:609:DOD:D2[2_555]	1.54	0.66

5.3 Torsion angles (i)

5.3.1 Protein backbone (i)

In the following table, the Percentiles column shows the percent Ramachandran outliers of the chain as a percentile score with respect to all X-ray entries followed by that with respect to entries of similar resolution.

The Analysed column shows the number of residues for which the backbone conformation was analysed, and the total number of residues.

Mol	Chain	Analysed	Favoured	Allowed	Outliers	Percentiles
1	A	604/306 (197%)	584 (97%)	20 (3%)	0	100 100

There are no Ramachandran outliers to report.

5.3.2 Protein sidechains (i)

In the following table, the Percentiles column shows the percent sidechain outliers of the chain as a percentile score with respect to all X-ray entries followed by that with respect to entries of similar resolution.

The Analysed column shows the number of residues for which the sidechain conformation was analysed, and the total number of residues.

Mol	Chain	Analysed	Rotameric	Outliers	Percentiles
1	A	524/263 (199%)	518 (99%)	6 (1%)	73 65

All (6) residues with a non-rotameric sidechain are listed below:

Mol	Chain	Res	Type
1	A	27[A]	LEU
1	A	27[B]	LEU
1	A	59[A]	ILE
1	A	59[B]	ILE
1	A	235[A]	MET

Continued on next page...

CONFIDENTIAL VALIDATION REPORT

Continued from previous page...

Mol	Chain	Res	Type
1	A	235[B]	MET

Sometimes sidechains can be flipped to improve hydrogen bonding and reduce clashes. There are no such sidechains identified.

5.3.3 RNA (i)

There are no RNA molecules in this entry.

5.4 Non-standard residues in protein, DNA, RNA chains (i)

There are no non-standard protein/DNA/RNA residues in this entry.

5.5 Carbohydrates (i)

There are no monosaccharides in this entry.

5.6 Ligand geometry (i)

1 ligand is modelled in this entry.

In the following table, the Counts columns list the number of bonds (or angles) for which Mogul statistics could be retrieved, the number of bonds (or angles) that are observed in the model and the number of bonds (or angles) that are defined in the Chemical Component Dictionary. The Link column lists molecule types, if any, to which the group is linked. The Z score for a bond length (or angle) is the number of standard deviations the observed value is removed from the expected value. A bond length (or angle) with $|Z| > 2$ is considered an outlier worth inspection. RMSZ is the root-mean-square of all Z scores of the bond lengths (or angles).

Mol	Type	Chain	Res	Link	Bond lengths			Bond angles		
					Counts	RMSZ	$\#\|Z\| > 2$	Counts	RMSZ	$\#\|Z\| > 2$
2	I1W	A	401	1	47,49,49	1.15	3 (6%)	59,77,77	1.51	13 (22%)

In the following table, the Chirals column lists the number of chiral outliers, the number of chiral centers analysed, the number of these observed in the model and the number defined in the Chemical Component Dictionary. Similar counts are reported in the Torsion and Rings columns. '-' means no outliers of that kind were identified.

Mol	Type	Chain	Res	Link	Chirals	Torsions	Rings
2	I1W	A	401	1	-	1/39/78/78	0/5/5/5

CONFIDENTIAL VALIDATION REPORT

All (3) bond length outliers are listed below:

Mol	Chain	Res	Type	Atoms	Z	Observed(Å)	Ideal(Å)
2	A	401	I1W	C32-C31	3.03	1.60	1.56
2	A	401	I1W	C7-C6	2.50	1.42	1.36
2	A	401	I1W	C8-C9	2.46	1.42	1.36

All (13) bond angle outliers are listed below:

Mol	Chain	Res	Type	Atoms	Z	Observed(^{o})	Ideal(^{o})
2	A	401	I1W	C30-C31-N36	-4.67	102.25	107.34
2	A	401	I1W	C3-C2-C18	-3.53	107.36	111.31
2	A	401	I1W	C32-C31-C30	2.98	117.22	113.40
2	A	401	I1W	C32-C31-N36	2.93	115.51	111.84
2	A	401	I1W	C28-C27-N26	2.89	121.04	115.57
2	A	401	I1W	C10-C5-N4	2.81	114.59	108.04
2	A	401	I1W	C33-C32-C31	2.51	114.78	109.70
2	A	401	I1W	C39-N38-C37	2.46	128.75	125.37
2	A	401	I1W	C18-N26-C27	2.46	127.42	123.07
2	A	401	I1W	C49-C1B-C46	-2.38	58.73	60.15
2	A	401	I1W	C5-C10-S11	-2.26	108.85	111.85
2	A	401	I1W	C19-C18-N26	-2.07	107.50	110.18
2	A	401	I1W	O50-C27-N26	-2.03	119.17	122.93

There are no chirality outliers.

All (1) torsion outliers are listed below:

Mol	Chain	Res	Type	Atoms
2	A	401	I1W	N26-C18-C2-O1

There are no ring outliers.

1 monomer is involved in 2 short contacts:

Mol	Chain	Res	Type	Clashes	Symm-Clashes
2	A	401	I1W	2	0

The following is a two-dimensional graphical depiction of Mogul quality analysis of bond lengths, bond angles, torsion angles, and ring geometry for all instances of the Ligand of Interest. In addition, ligands with molecular weight > 250 and outliers as shown on the validation Tables will also be included. For torsion angles, if less then 5% of the Mogul distribution of torsion angles is within 10 degrees of the torsion angle in question, then that torsion angle is considered an outlier. Any bond that is central to one or more torsion angles identified as an outlier by Mogul will be highlighted in the graph. For rings, the root-mean-square deviation (RMSD) between the ring

CONFIDENTIAL VALIDATION REPORT

in question and similar rings identified by Mogul is calculated over all ring torsion angles. If the average RMSD is greater than 60 degrees and the minimal RMSD between the ring in question and any Mogul-identified rings is also greater than 60 degrees, then that ring is considered an outlier. The outliers are highlighted in purple. The color gray indicates Mogul did not find sufficient equivalents in the CSD to analyse the geometry.

Ligand I1W A 401

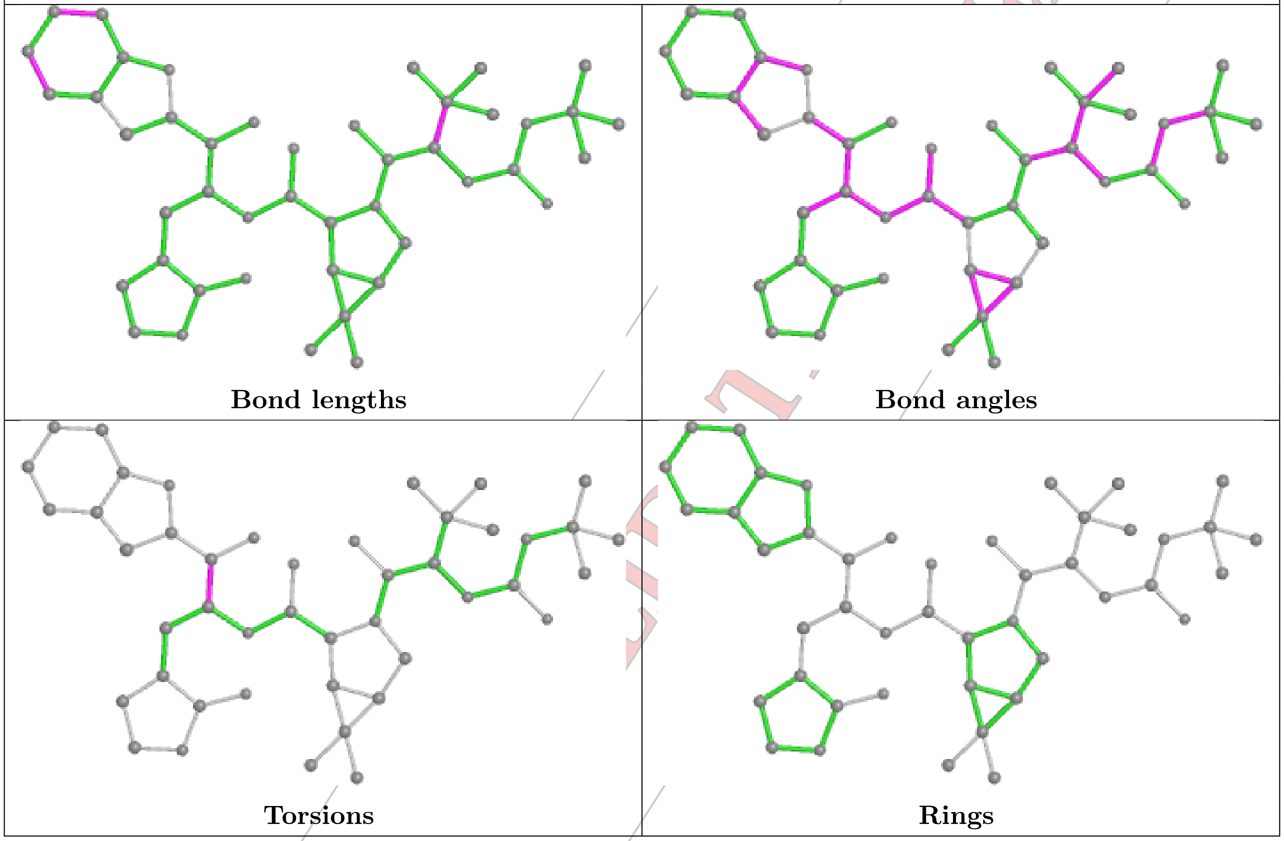

5.7 Other polymers (i)

There are no such residues in this entry.

5.8 Polymer linkage issues (i)

There are no chain breaks in this entry.

CONFIDENTIAL

6 Fit of model and data (i)

6.1 Protein, DNA and RNA chains (i)

EDS failed to run properly - this section is therefore empty.

6.2 Non-standard residues in protein, DNA, RNA chains (i)

EDS failed to run properly - this section is therefore empty.

6.3 Carbohydrates (i)

EDS failed to run properly - this section is therefore empty.

6.4 Ligands (i)

EDS failed to run properly - this section is therefore empty.

6.5 Other polymers (i)

EDS failed to run properly - this section is therefore empty.

CONFIDENTIAL VALIDATION REPORT
